# Unique *In Vitro* and *In Vivo* Thrombopoietic Activities of Ingenol 3,20 Dibenzoate, A Ca^++^-Independent Protein Kinase C Isoform Agonist

**DOI:** 10.1371/journal.pone.0051059

**Published:** 2012-12-21

**Authors:** Frederick K. Racke, Maureen Baird, Rolf F. Barth, Tianyao Huo, Weilian Yang, Nilendu Gupta, Michael Weldon, Heather Rutledge

**Affiliations:** 1 Department of Pathology, The Ohio State University School of Medicine, Columbus, Ohio, United States of America; 2 Department of Radiation Oncology, The Ohio State University School of Medicine, Columbus, Ohio, United States of America; University of Granada, Spain

## Abstract

Thrombopoiesis following severe bone marrow injury frequently is delayed, thereby resulting in life-threatening thrombocytopenia for which there are limited treatment options. The reasons for these delays in recovery are not well understood. Protein kinase C (PKC) agonists promote megakaryocyte differentiation in leukemia cell lines and primary cells. However, little is known about the megakaryopoietic effects of PKC agonists on primary CD34+ cells grown in culture or *in vivo*. Here we present evidence that the novel PKC isoform-selective agonist 3,20 ingenol dibenzoate (IDB) potently stimulates early megakaryopoiesis of human CD34+ cells. In contrast, broad spectrum PKC agonists failed to do so. *In vivo*, a single intraperitoneal injection of IDB selectively increased platelets in mice without affecting hemoglobin or white counts. Finally, IDB strongly mitigated radiation-induced thrombocytopenia, even when administered 24 hours after irradiation. Our data demonstrate that novel PKC isoform agonists such as IDB may represent a unique therapeutic strategy for accelerating the recovery of platelet counts following severe marrow injury.

## Introduction

Despite recent advances in our understanding of megakaryocyte growth and platelet production, thrombocytopenia remains a difficult problem in the clinical management of patients with hematologic malignancies. Thrombopoietin (TPO) is the major cytokine involved in the normal production of platelets [1], [2]. However, TPO has been relatively unsuccessful in the treatment of these patients, particularly in shortening the “thrombocytopenic window” following therapy [3], [4], [5], [6]. Thus, platelet transfusions remain the primary treatment for thrombocytopenia despite its significant costs and relatively short-lived responses [7]. Because of this, there remains an important clinical need for the development of novel approaches to accelerate platelet recovery following myeloablative therapy or severe marrow injury. It has been over 25 years since the original observations were reported describing the unique ability of protein kinase C (PKC) agonists to promote megakaryocytic differentiation of primary murine cells [8], [9]. However, these early studies, which examined the effects of PKC agonists on megakaryocytic differentiation of normal hematopoietic progenitors, were carried out before megakaryocyte culture conditions from early CD34+ progenitors had been developed. Furthermore, these early studies were done prior to the cloning of TPO, which has markedly improved the ability to generate megakaryocytes from hematopoietic progenitors *in vitro*. Despite this, hundreds of reports have been published in which PKC agonists have been utilized to study various aspects of megakaryocyte differentiation, primarily in leukemia cell lines with megakaryocytic potential [10], [11]. However, little is known about the megakaryopoietic effects of PKC agonists on primary CD34-selected cells grown in well-defined culture conditions or *in vivo*.

Beyond the early hematopoietic precursor stages, one of the late decision points in hematopoietic lineage separation is the bifurcation of erythroid and megakaryocytic lineages from a common precursor, the megakaryocytic-erythroid progenitor (MEP) [12]. The growth factors most responsible for megakaryocytic and erythroid development, TPO and erythropoietin (EPO), respectively, share significant sequence homology [2] and activate many of the same signal transduction pathways [13]. Nevertheless, there is little, if any, reciprocal lineage infidelity during either normal erythroid or megakaryocytic differentiation so that cultures of CD34+ progenitors in EPO gives rise to a relatively pure population of erythroblasts and culture in TPO supports virtually no erythroid growth. The discriminating elements for this divergent differentiation scheme are unknown. One potential candidate is Protein Kinase C (PKC). This is a heterogeneous family of serine/threonine kinases initially described as a calcium and phospholipid dependent kinase, which functions as the cellular receptor for phorbol esters [14]. Numerous studies have shown individual PKC isoforms have distinct and non-overlapping roles in cellular function [15], [16], [17], [18]. There is also evidence of their redundancy to regulate certain biological functions such as ERK activation [15], [16], [17], [18]. Important roles for various PKC isoforms have been demonstrated in the differentiation of a variety hematopoietic lineages. For example, the PKCα isoform appears to be required for EPO-induced erythroid differentiation [19], as well as macrophage development from bipotential granulocyte-macrophage colony-forming cells [20]. Conversely, PKCε expression was found to be down-regulated during early erythroid differentiation [19]. One of us (FKR) previously has reported that PKCε was upregulated during megakaryocytic differentiation of K562 cells and that it cooperated functionally with GATA-1 to activate the megakaryocyte specific αIIb promoter [21]. In addition, it was observed that the novel PKC isoform-selective agonist ingenol 3,20 dibenzoate (IDB) induced megakaryocytic differentiation of K562 cells, as well as the selective translocation of PKCε. Together, these data suggest that novel PKC isoforms, particularly PKCε, may play a unique role in the regulation of megakaryocytic differentiation.

In the present study, we have characterized the effects of IDB on the growth and differentiation of CD34+ progenitors and its effects on thrombopoiesis *in vivo*. IDB has distinct activity to promote early megakaryocytic differentiation of primary human CD34+ hematopoietic cells and to stimulate the early expression of transcription factors known to participate in the megakaryocytic differentiation. Finally, and perhaps most importantly, IDB stimulated megakaryocyte and platelet production in mice and strongly mitigated radiation-induced thrombocytopenia. Our data suggest that IDB, a PKCε agonist, may represent a novel thrombopoietic drug for the treatment of thrombocytopenia.

## Results

### Ingenol 3,20 dibenzoate promotes early megakaryocytic differentiation of CD34+ human hematopoietic progenitors whereas other PKC agonists do not

**Figure 1:**
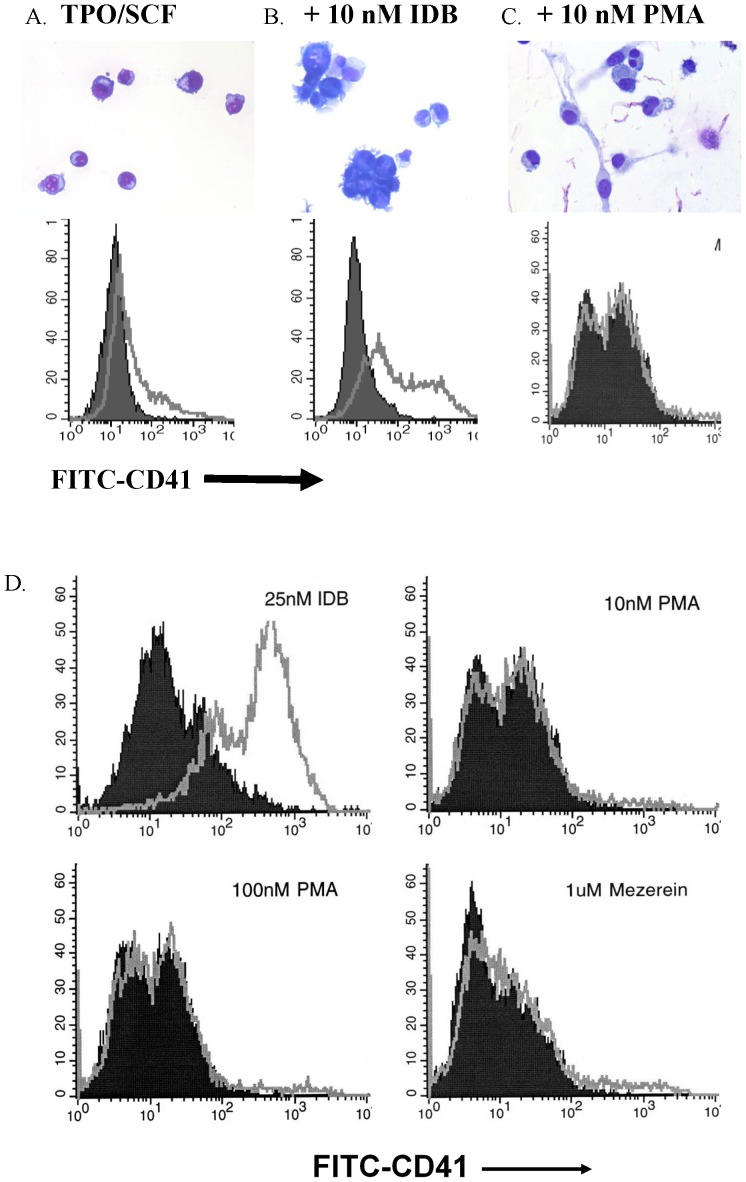
IDB induces megakaryocytic differentiation of human CD34+ progenitors whereas PMA does not. **(A–C, upper panels)** Morphology of adult human CD34+ hematopoietic progenitors cultured for 7 days in medium containing TPO and SCF alone or with 10 nM IDB or 10 nM PMA on Wright-stained preparations. Note that relative to control cells, cells treated with IDB were larger and had polyploid nuclei. **(A–C, lower panels)** Adult human CD34+ progenitors were treated with TPO and SCF with or without 10 nM IDB or 10 nM PMA. After 7 days of culture, the cells were stained with a fluorescent antibody directed against CD41a (open profiles) or with an isotype matched control antibody (black profiles). All flow cytograms in this and Figures 2 and 3 are representative of at least 3 independent runs. (D) Adult human CD34+ progenitors were treated with TPO and SCF with 25 nM IDB, 10 nM PMA, 100 nM PMA, or 1 µM Mezerein. After 7 days of culture, the cells were stained with a fluorescent antibody directed against CD41a (open profiles) or with an isotype matched control antibody (black profiles).

CD34+ progenitor cells, while morphologically immature, contain a mixture of hematopoietic progenitors with varying degrees of lineage commitment. There are relatively few committed megakaryocytic progenitors in the CD34+ population. In order to generate large numbers of megakaryocytes, TPO must be combined with another cytokine that supports the growth and survival of more primitive progenitors. There is increasing experimental evidence that these earlier progenitors are MEPs [12], [22], [23] that give rise to both erythroid and megakaryocytic cells. Culturing CD34+ progenitors in TPO and stem cell factor (SCF) produced large numbers of megakaryocytic cells without significant contamination of erythroid cells. However, when CD34+ progenitors were cultured in TPO/SCF, the early period of culture was characterized by proliferation with little terminal megakaryocytic differentiation, typically occurring in the first week in culture (Fig. 1A). Since PKC agonists promote megakaryocytic differentiation of erythroleukemia cell lines, we investigated whether they might promote early megakaryocytic differentiation of normal human CD34+ cells. It was noteworthy that the addition of IDB to TPO/SCF-containing cultures promoted early megakaryocyte differentiation (Fig. 1B), whereas the addition of the broad spectrum PKC agonist phorbol myristate acetate (PMA) did not (Fig. 1C). Based on 3 independent runs of each sample, these findings were confirmed by flow cytometric analysis using FITC-CD41 antibody (independent runs of each sample). Varying concentrations of PMA or the addition of other non-phorbol broad spectrum PKC agonists, including mezerein (Fig. 1D) and indolactam V (data not shown) also failed to induce early megakaryocyte differentiation. The increase in megakaryocytes in IDB-containing cultures was not due to a selective inhibition of growth of non-megakaryocytic cells since cell proliferation studies showed that IDB did not reduce proliferation over the same time period in cultures stimulated by TPO/SCF, EPO/SCF, or GM-CSF/SCF (data not shown). Thus, under liquid culture conditions, optimized for megakaryocytic growth, non-selective PKC agonists failed to promote early megakaryocytic differentiation whereas IDB promoted early megakaryocytic differentiation.

### IDB induces early expression of markers of megakaryocytic differentiation

During normal megakaryocyte differentiation, acquisition of the tetraspanin molecule CD9 has been identified as an early surface marker of megakaryocyte differentiation [24]. While its expression is not limited to megakaryocytes, it is not expressed on erythroid cells and thus its expression marks the erythromegakaryocytic bifurcation [25]. Previous studies have shown that the CD9 promoter possesses PKC responsiveness and CD9 can be induced in leukemic cell lines induced by PKC agonists to undergo megakaryocytic differentiation [26].

**Figure 2:**
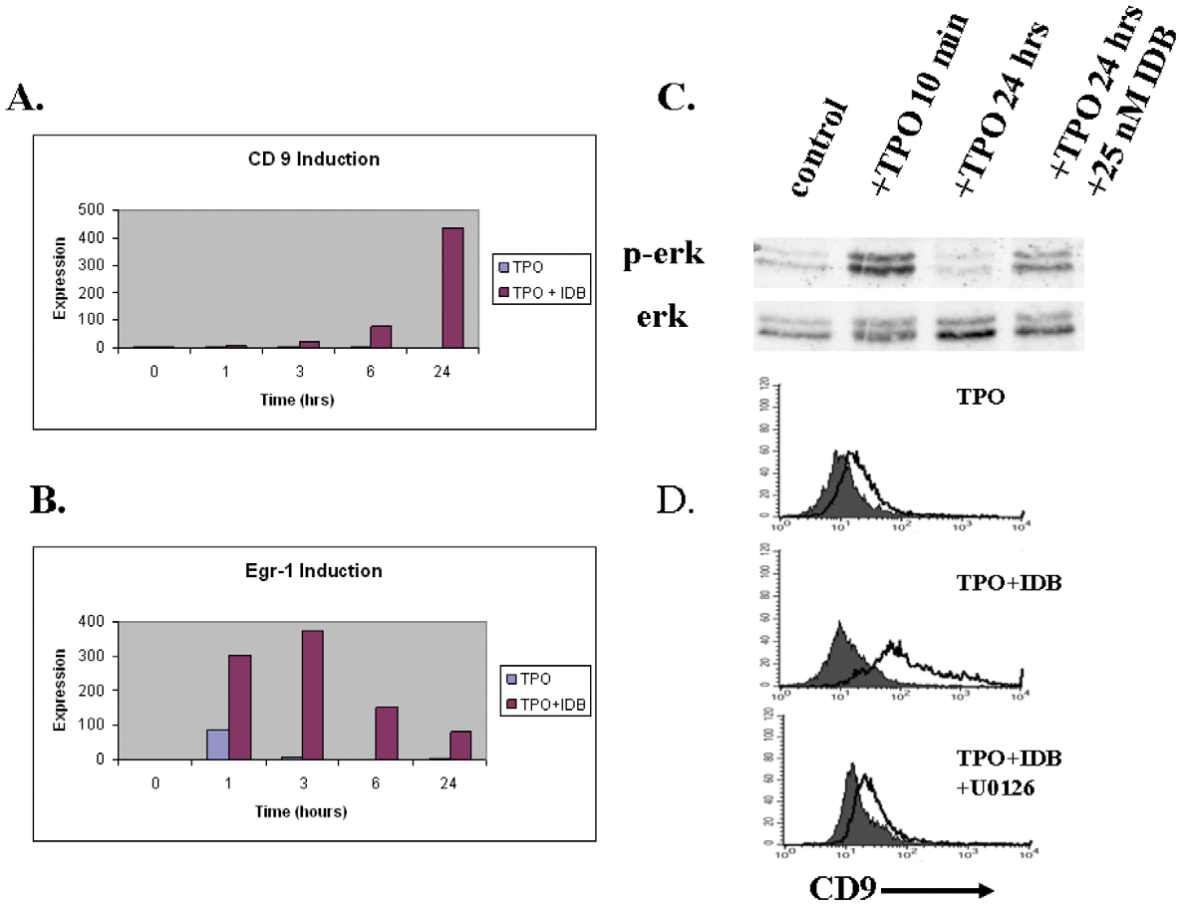
IDB induction of ERK activation, egr-1, and CD9 in CD34+ cells. (**A&B**) CD34+ cells were thawed and recovered overnight in serum-free medium supplemented with SCF (25 ng/ml). Cells were stimulated with TPO (40 ng/ml) with 25 nM IDB or vehicle. mRNA for CD9 and egr-1 were quantitated using SYBR green and real time PCR. The values shown in A and B represent the mean of 2–3 replicate determinations. (**C**) CD34+ cells were recovered overnight as described above. Cells were stimulated with TPO with or without IDB as indicated. Immunoblot analysis of phospho-ERK and total ERK then were performed on whole cell lysates. (**D**) CD34+ cells were cultured in serum-free media and TPO/SCF with or without 100 nM IDB+/− 10 µM of the MEK inhibitor U0126 for 3 days and then analyzed for CD9 expression by flow cytometry.

**Figure 3:**
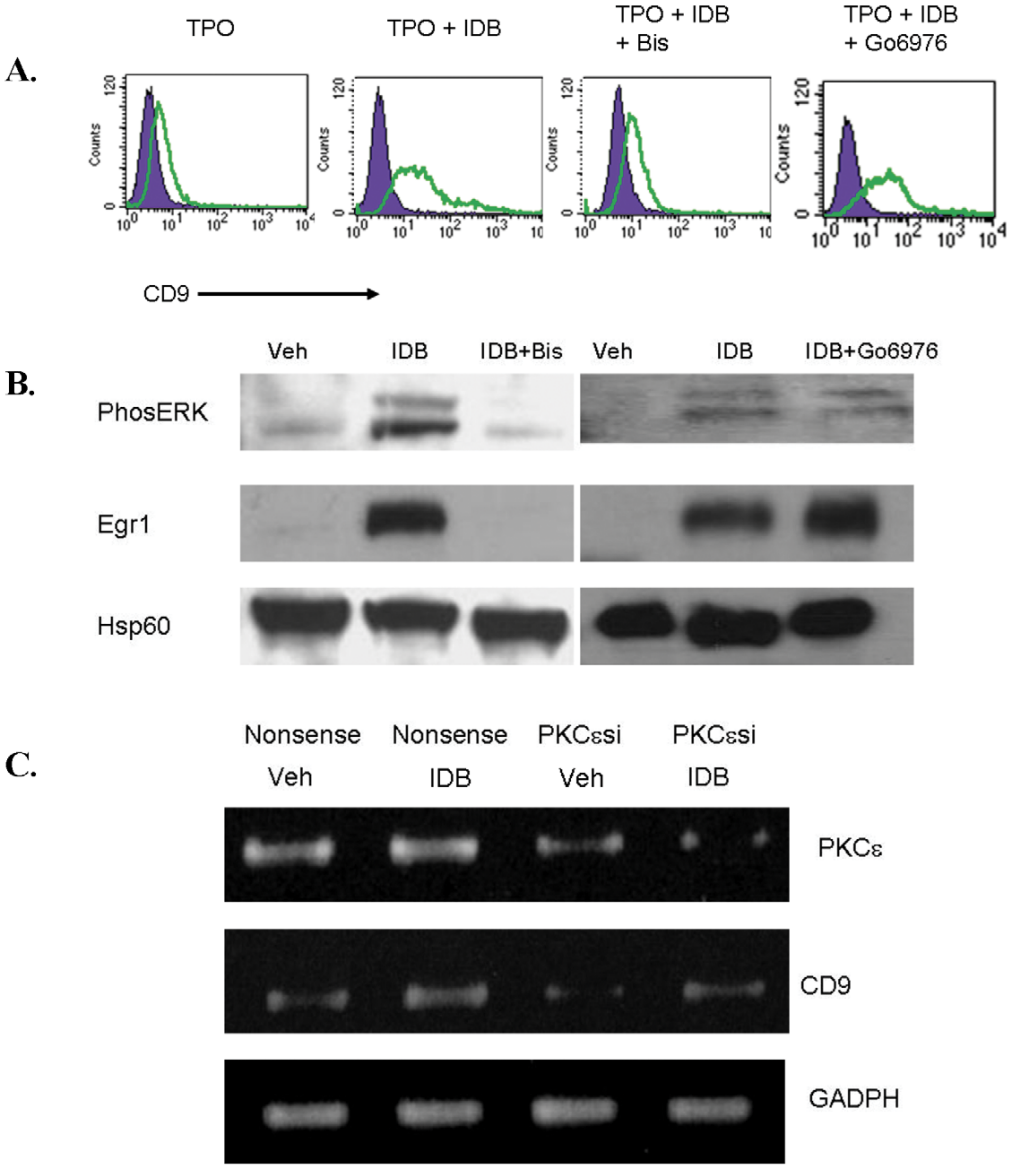
Inhibitor studies support that CD9 induction by IDB is mediated by PKCε. (**A**) CD34+ cells were cultured in TPO/SCF for 3 days with or without 100 nM IDB +/− 5 µM of GF109203X or 1 µM Go6976 and analyzed for CD9 (open panel) or isotype control (shaded panel) by flow cytometry. (**B**) CD34+ cells were cultured in TPO/SCF for 2 hours with or without 100 nM IDB +/− GF109203X or Go6976 and subjected to western blot analysis for the indicated antigens. (**C**) CD34+ cells were cultured for 48 hours in media containing TPO/SCF. Cells were then nucleofected as per the manufacturer's protocol for CD34+ cells with either scrambled or PKCε siRNA. After a 2 hour recovery, the cells were treated with either vehicle or 25 nM IDB for an additional 24 hours and mRNA was extracted and analyzed by PCR for CD9, PKCε, and GAPDH expression.

In order to understand the early events associated with IDB-induced megakaryocytic differentiation in primary cells, we examined the early expression of CD9. Induction of CD9 expression at the mRNA level was seen within the first 24 hours of exposure to IDB (Fig. 2A). We and others have shown that PKC activation in K562 erythroleukemia cells induced sustained activation of the MAP kinase ERK [27], [28]. IDB also increased the expression of Egr-1 (Fig. 2B) and induced prolonged ERK activation of CD34+ cells (Fig. 2C). Previous studies have shown that the expression levels of the immediate early gene egr-1 was exquisitely sensitive to the amplitude and duration of the ERK activity [29]. In addition, egr-1 has been implicated in the regulation a number of megakaryocyte expressed genes. As previously reported by Racke et al. egr-1 is involved in the regulation of CD9 expression in IDB treated K562 cells [30]. Inhibition of ERK activity by the MEK inhibitor U0126 blocked the induction of CD9 expression by IDB (Fig. 2D). CD9 induction was completely abrogated by GF109203X (Fig. 3A), a broad spectrum inhibitor of both classical and novel PKC isoforms. GF109203X also inhibited ERK activation and egr-1 induction (Fig. 3B). In contrast, Go6976, which inhibits the classical PKC isoforms but not the novel isoforms, failed to block CD9 or egr-1 induction by IDB. Finally, knockdown of PKCε by siRNA in CD34+ cells reduced CD9 induction (Fig. 3C). Together, these data suggest that IDB induced sustained ERK activation by novel PKC isoforms, and specifically PKCε in CD34+ cells. This activation is important for the induction of the early megakaryocyte marker CD9.

**Figure 4:**
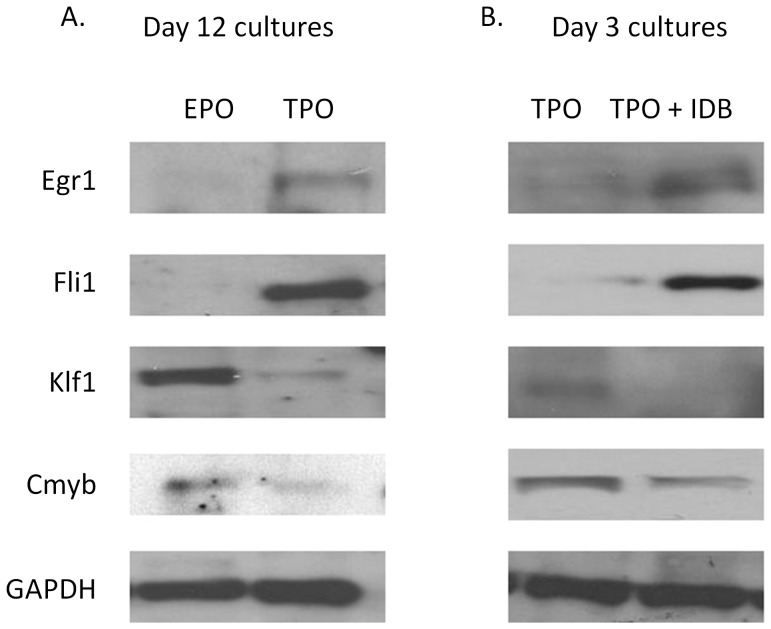
IDB induces early expression of promegakaryopoietic transcription factors. (**A**) CD34+ cells cultured in TPO/SCF or EPO/SCF for 12 days and subjected to Western blot analysis. (**B**)TPO/SCF +/− 100 nM IDB for 72 hrs and subjected to western blot analysis. Blots were then stripped and reprobed for each of the indicated proteins.

Recently, several reports have shown that the balance of the levels of lineage-defining transcription factors such as c-myb, eklf, and Fli-1 may have critical effects on the balance of erythroid and megakaryocytic differentiation. To further evaluate the events triggered by IDB in early megakaryocytic differentiation of CD34+ cells, we evaluated the expression of these factors. Both c-myb and eklf have been shown to be critical for erythroid differentiation [31], [32], [33]. Fli-1, on the other hand, appears important for megakaryocytic differentiation [34], [35]. Importantly, fli-1 and eklf have been shown to possess cross-antagonism in the control of the erythromegakaryocytic bifurcation [36]. Consistent with their reported roles in erythroid differentiation c-myb and eklf were expressed in erythroid cultures, whereas fli-1 and egr-1 were detectable in megakaryocytic cultures (Fig. 4A). Profiling the expression of these transcription factors in early day 3 cultures with TPO/SCF revealed that there was relatively little expression of the megakaryocytic transcription factors egr-1 and fli-1 (Fig. 4B). However, addition of IDB resulted in a strong induction of egr-1 and fli-1 without a similar increase in klf-1 or c-myb (Fig. 4B).

### IDB promotes thrombopoiesis *in vivo*

**Figure 5:**
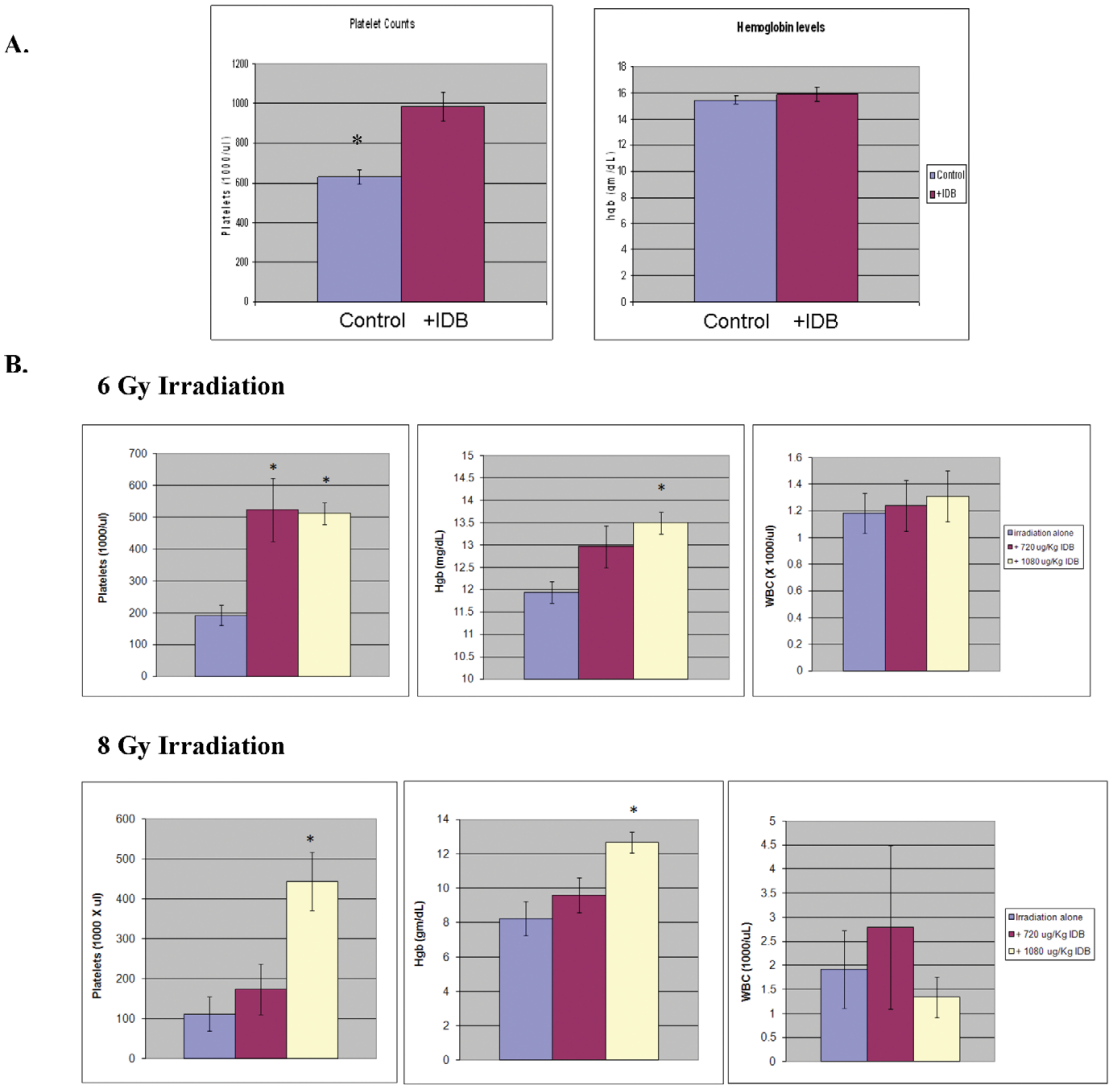
Single i.p. injection of IDB increases platelet counts and mitigates thrombocytpoenia induced by irradiation. (**A**) A single i.p. injection of IDB increased platelet counts at day 7 compared with vehicle injected control animals (N = 5 per group; *p<0.01). No differences were observed in hemoglobin and white blood counts (p = NS). (**B**) Groups of BALB/c mice (N = 10/group/time point) were injected with a single i.p. dose of IDB (720 or 1080 µg/kg body weight) 3 hours before irradiation with 6 or 8 Gy. At two weeks the mice treated with 6 Gy were euthanized and complete blood counts were performed. At three weeks the mice treated with 8 Gy were euthanized and complete blood counts were taken (* showed statistical significance of at least p<0.01 or greater).

**Figure 6:**
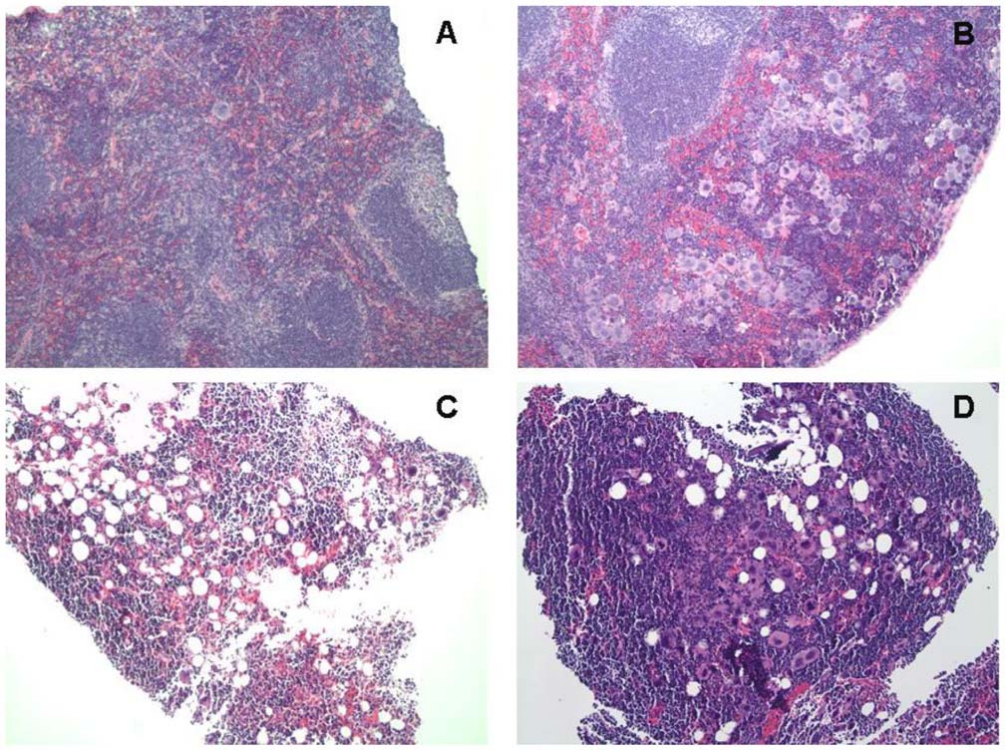
IDB increases post-irradiation megakaryocyte production in both the spleen and bone marrow. Histologic sections of spleens (upper panels) or bone marrow (lower panels) 2 weeks after mice received 6 Gy irradiation alone (**A** and **C**) or 6Gy + 1080 µg/kg IDB 3 hours prior to irradiation (**B** and **D**).

**Figure 7:**
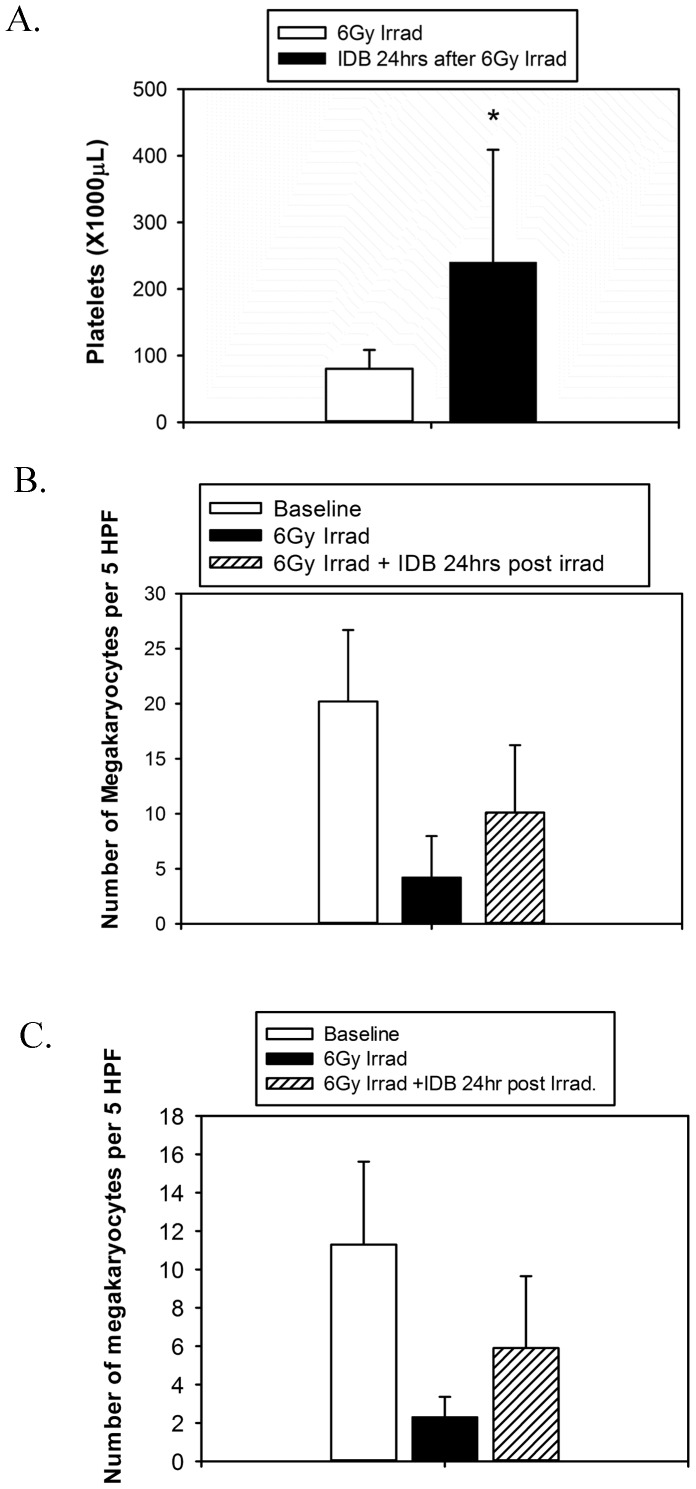
Single i.p. injection of IDB increases platelet counts and megakaryocyte content even when administered 24 hours after irradiation. Groups of BALB/c mice (N = 10/group/time point) were injected with a single i.p. dose of IDB (1080 µg/kg body weight) 24 hours after irradiation with 6 Gy. At 2 weeks, mice were euthanized and blood platelet counts (**A**) were performed and tissue for histologic examination for megakaryocyte content of bone marrow (**B**) and spleen (**C**) were obtained. Megakaryocyte baseline values were determined on 5 untreated mice, euthanized to obtain spleens and bone marrows for histologic examination.

**Figure 8:**
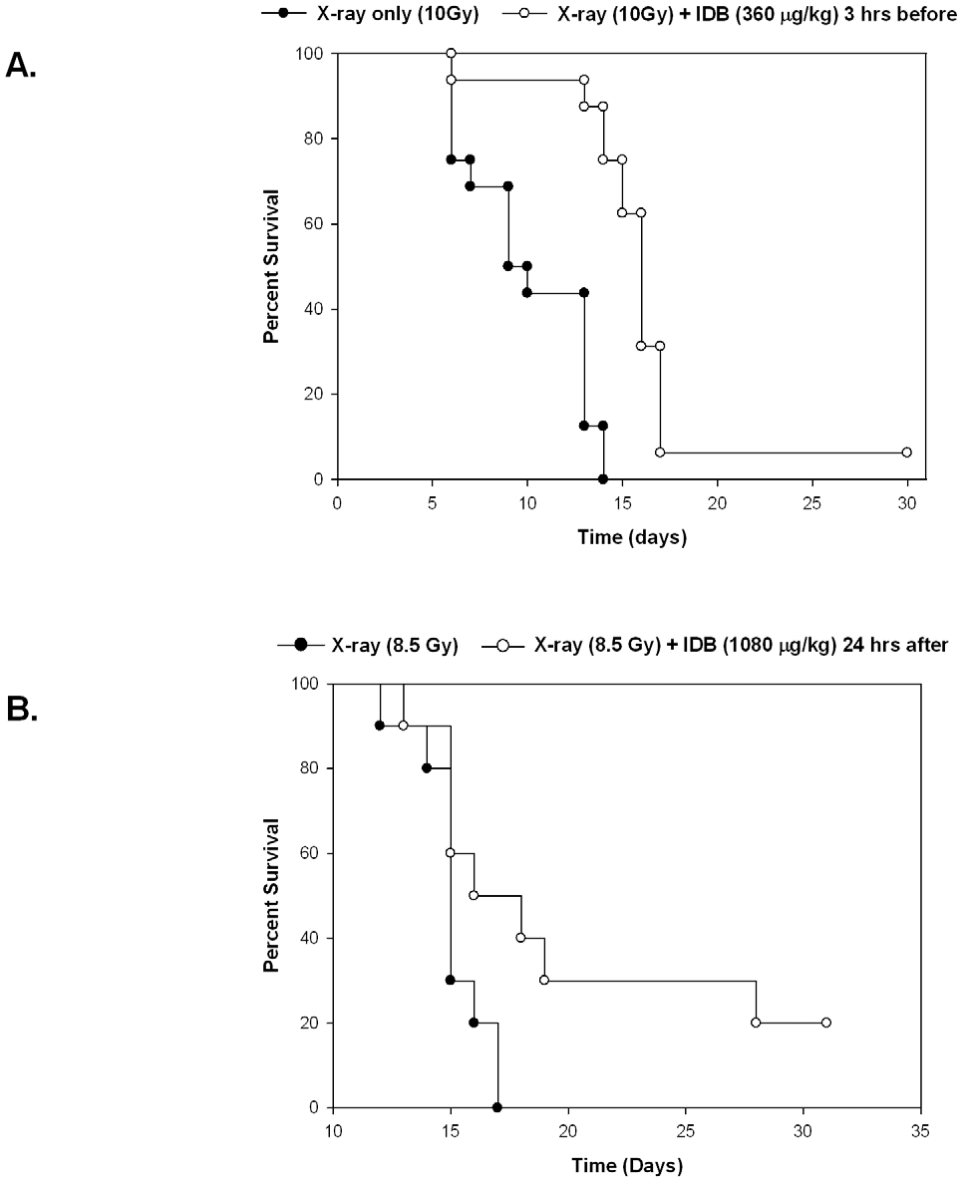
IDB improves survival of lethally irradiated mice following IDB administration 3 hours prior to or 24 hours after irradiation. (**A**) The survival of BALB/c mice was significantly improved **(**p-value<0.001 using Log-rank test) when they received IDB (360 µg/kg b.w.) 3 hours before 10.0 Gy X-ray (6 MV photon) compared to the survival of the mice received X-ray only. (**B**)The survival of BALB/c mice was significantly improved **(**p-value = 0.0289, using the Log-rank test) when they received 8.5 Gy X-ray (6 MV) followed by IDB (1080 µg/kg b.w.) 24 hours later, compared to the survival of the mice received X-irradiation without IDB.

Because of the observed effects of IDB on the early *in vitro* production of megakaryocytes, we investigated the *in vivo* effects on megakaryopoiesis and platelet production. In control mice, a single i.p. injection of IDB led to dose-dependent increases in platelet counts to 50% above baseline at 1 week and had no effect on hemoglobin (Fig. 5A). Gross examination of organs and cavities of mice treated with IDB did not show any obvious signs of inflammation, suggesting that the thrombopoietic effect of IDB was not secondary to an inflammatory response to IDB. Next, in order to investigate whether IDB could stimulate early platelet recovery, we employed a murine model of radiation-induced thrombocytopenia (Fig. 5A). Mice treated with low dose radiation (2–4 Gy) had a transient drop in both platelet and WBC counts. Pretreatment with IDB 3 hrs prior to irradiation increased the platelet counts without improving WBC (data not shown). Higher radiation doses (6 or 8 Gy) caused prolonged pancytopenia. IDB treatment 3 hrs prior to either 6 or 8 Gy X-irradiation significantly reduced the thrombocytopenia (Fig. 5B; p<0.005) without affecting the drop in WBC (Fig. 5B; p = NS) at 14 and 21 days respectively following irradiation. Unexpectedly, radiation-induced anemia also was mitigated with hemoglobin values of 12.7±2.0 mg/dL for mice that received an 8 Gy X-ray dose plus 1080 µg/kg IDB prior to irradiation compared to 8.2±3.1 mg/dL for those that did not and 13.5±0.7 vs. 11.9±0.8 mg/dL, respectively, for mice that received a dose of 6 Gy (Fig. 5B; p<0.01). One possible explanation for this might be that improved hemostasis due to the increased platelet counts resulted in less bleeding and improved hemoglobin levels. Since hematocrits were not determined, we cannot exclude the possibility that there may have been an increase in cellular hemoglobin without an increase in RBC numbers. This improvement in platelet counts was accompanied by a substantial increase in the megakaryocyte content of hematopoietic compartments (Fig. 6). As shown in Fig. 7A IDB mitigated radiation-induced thrombocytopenia, even when administered 24 hrs after irradiation. Platelet counts increased from 240.5±169.1 (×1000/µL) for mice that received a 6 Gy X-ray dose plus IDB 24 hrs post irradiation compared to 80.3±28.2 (×1000/µL) (P = 0.008) for those that did not. The improvement in platelet counts again was accompanied by increases in megakaryocytes (Fig. 7B) in both bone marrow from 4.2±3.8 to 10.1±6.1 megakaryocytes per 5 high power field (hpf) (Fig. 7B, P<0.01), and spleens from 2.3±1.1 to 5.9±3.8 megakaryocytes per 5 hpf(Fig. 7C, P<0.001) compared to untreated irradiated controls. The mean survival times (MSTs) of those mice that received 360 µg/kg of IDB 3 hrs prior to X-irradiation with 10 Gy was 15.9±4.6 d compared to 10.1±3.2 d for untreated controls. Animals that received IDB (1080 µg/kg) 24 hrs after X-irradiation with 8.5 Gy had a MST of 20.1±7.1 d compared to 15.1±1.5 d for untreated controls. Finally, as shown in Fig. 8, IDB significantly improved the MSTs of lethally irradiated mice, when administered 3 hours prior or 24 h following X-irradiation. The differences in the Kaplan Meier survival plots for mice that were irradiated 3 hours after administration of IDB was P<0.001 and P = 0.0289 for those treated with IDB 24 hrs following radiation compared to untreated controls. These data demonstrate that the effects of IDB to enhance megakaryocytic production *in vitro* resulted in early megakaryocyte recovery and increased platelet production *in vivo* following serious marrow injury.

## Discussion

Bone Marrow recovery following myeloablative therapy or bone marrow transplantation is almost always accompanied by prolonged thrombocytopenia necessitating platelet transfusions to prevent life-threatening bleeding. This so-called “thrombocytopenic window” generally lasts for 2–3 weeks following myeloablation. Earlier studies failed to demonstrate an effect of TPO to shorten this window [3], [4], [5], [6]. Platelet recovery under such conditions involves megakaryocyte reconstitution from early hematopoietic progenitors, which reside in the CD34+ compartment of the bone marrow. Among these are the bipotent MEPs which give rise to erythroid cells and megakaryocytes. Previous studies, demonstrating the presence of MEPs using single cell colony assays, showed that the composition of the colonies derived from MEPs was heavily weighted towards erythroid cells. There was up to a 100–1000 fold increase in erythroblasts but only a small number of megakaryocytes, indicating a natural bias towards erythropoiesis [12]. Thus, it is feasible that agents that might promote early megakaryocyte differentiation from CD34+ cells may be able to facilitate early megakaryocyte and platelet reconstitution following severe marrow injury.

We have shown that the addition of IDB increased the production of megakaryocytes early in CD34+ cell cultures. This effect was not observed following the addition of broad spectrum PKC agonists, which seemed to have an inhibitory effect on proliferation and on megakaryocytic differentiation. Because broad spectrum PKC agonists such as phorbol ester also activate the novel PKC isoforms, the reason for this difference is unclear. Since, in contrast to IDB, phorbol esters activate both the classical and the novel PKC isoforms, one possible explanation is that activation of classical isoforms antagonizes the megakaryopoietic potential of early progenitors. Classical PKC isoforms, and specifically PKCβII, have been shown to promote dendritic cell differentiation of CD34+ cells and PMA applied to CD34+ cells induced approximately half of the cellular input to undergo dendritic differentiation by day 7 [37], [38]. Although we did not analyze our CD34+ cultures for dendritic markers, the morphology of the cells treated with PMA appears consistent with these reported results.

A potential role for PKCε in regulating the erythromegakaryocytic bifurcation has been suggested in previous work. First, PKCε expression is induced in early megakaryocytic differentiation and reduced in early erythroid differentiation [39]. Peptide inhibitors of PKCε have been shown to enhance erythroid colony formation two-fold [40]. Interestingly, in later erythroid maturation PKCε is re-expressed and appears to protect late erythroblasts from TRAIL-induced apoptosis [41]. Conversely, PKCε is down-regulated in late megakaryocyte maturation and over expression of PKCε in maturing megakaryocytes appears to block terminal maturation [39]. Thus, it appears that PKC expression is dynamic and the timing of activation may be an important factor in determining its effects on differentiation. Our data using both agonists and antagonists support our hypothesis that agonists selective for novel PKC isoforms, and particularly PKCε, have enhanced activity to promote early megakaryocytic differentiation from normal CD34+ hematopoietic progenitors and offer a unique therapeutic approach to promoting early megakaryocyte and platelet recovery following marrow injury.

While controversy exists over whether cytokine signaling has instructive influences on hematopoietic differentiation, a number of recent studies have suggested that the balance of lineage-defining transcription factors may play a critical role in controlling the erythromegakaryocytic bifurcation. Mice bearing hypomorphic alleles of both p300 and c-myb have anemia and thrombocytosis [42], [43]. The authors of this study suggested that mechanisms to down-regulate the expression or inhibit the function of these transcription factors may represent novel strategies to promote platelet production. The data presented here suggest that IDB may serve as a tool to accomplish this, perhaps through expression of antagonizing megakaryocytic factors such as fli-1. Additional studies have shown that hypomorphic alleles of EKLF also result in anemia and thrombocytosis [44], [45]. In early cultures of CD34+ cells, the balance of transcription factors appears weighted towards erythropoiesis. This may be in part due to relative predominance in CD34+ cells already committed toward erythropoiesis. However, the composition of erythromegakaryocytic transcription factors in early cultures was strongly influenced by the addition of IDB. Thus, the shift in the balance of these lineage-defining transcription factors by IDB appears to be one potential mechanism that IDB promotes early megakaryocyte differentiation.

The effects of IDB to promote early megakaryocyte differentiation *in vitro* are mirrored by potent megakaryopoietic and thrombopoietic effects *in vivo*. In the murine model of radiation-induced thrombocytopenia that we have used, IDB strongly mitigated thrombocytopenia and shortened the thrombocytopenic window even when severe life-threatening levels of thrombocytopenia occurred. No protection was seen in the white blood counts, suggesting that this was not the result of a generalized protective effect on the bone marrow. Interestingly, while our *in vitro* data suggested that IDB would impair erythropoiesis, at higher doses of irradiation which caused significant anemia, we consistently observed an improvement in the hemoglobin levels in mice treated with IDB. It is possible that the improved hemostasis caused by the increased platelet counts reduced the blood loss related to internal bleeding. However, on the histologic sections there was no apparent significant decrease in the number of erythroid progenitors in the IDB treated mice following irradiation nor was there any evidence of intrasplenic congestion. Therefore, it is possible that there may have been an additional protective effect on erythroid precursors. As mentioned earlier, it has been reported that PKCε is re-expressed in later erythroid progenitors and may play a role in preventing apoptosis [41]. It is possible then that IDB protected committed erythroblasts from radiation-induced apoptosis. However, additional studies will be necessary to better define the *in vivo* effects of IDB on erythropoiesis.

The effectiveness of IDB to promote improved early megakaryocyte growth *in vitro* is supported by our *in vivo* data showing the improved early recovery of both platelet counts in the blood and megakaryocytes in the hematopoietic compartments following irradiation. IDB shortened the thrombocytopenic window, even when given up to 24 hours following irradiation. IDB given alone resulted in a modest but statistically significant improvement in survival of lethally-irradiated mice. Other factors, such as increased susceptibility to infections secondary to leukopenia, may have reduced the effectiveness of IDB to prolong survival time. In conclusion, our studies provide a conceptual framework to explain the cause of natural delay in megakaryocyte and platelet reconstitution following severe marrow injury. Furthermore, our data lay the groundwork for novel approaches to induce thrombopoiesis following severe radiation related marrow injury and suggest that IDB has the potential to succeed where thrombopoietin has failed. Therapies designed to influence erythromegakaryocytic lineage commitment may be clinically useful to promote early megakaryocyte and platelet reconstitution in patients with severe therapy-related marrow injury following myeloablative therapy for hematologic malignancies, bone marrow transplantation, or severe marrow injury such as accidental or intentional (i.e. terrorist related) exposures to irradiation. Finally, novel PKC agonists such as IDB could be useful tools to better understand early megakaryocyte and platelet recovery in these settings.

## Materials and Methods

### Cell Culture

Human primary CD34^+^ hematopoietic progenitor cells were purchased from the Cincinnati Children's Hospital. These were considered as existing material without any patient identifiers and therefore did not require Institutional Review Board approval for their use. They were purified from the peripheral blood of G-CSF treated normal adult donors and were grown in StemSpan SFEM serum free medium (Stem Cell Technologies, Vancouver, BC) supplemented with cytokines (R & D Laboratories, Minneapolis, MN). Cytokine concentrations were as follows: SCF (25 ng/ml), EPO (1 unit/ml), GM-CSF (5 ng/ml), and TPO (40 ng/ml) (R&D Systems, Minneapolis, MN). IDB (Alexis USA, San Diego, CA) was dissolved in dimethylsulfoxide (DMSO). For *in vivo* experiments, it was diluted with Phosphate Buffered Saline (PBS, pH 7.4) to yield the desired concentrations of IDB in 1% DMSO.

### Cell proliferation assay

CD34+ cells were transferred to 96 well microtiter plates at a concentration of 5,000 cells/100 µl. Following the addition of growth factors, the cells were placed into culture at 37°C and 5% CO_2_ for 0,3,or 6 days. After incubation, 10 µl of Cell Proliferation Agent WST-1 (Roche) was added to each well. Plates were then incubated again at 37°C for 0.5–4 hours. Following a brief agitation, absorbances were measured against a media control at 450 nM on a plate reader (Bio-Tek Instruments).

### Flow Cytometry

Staining of cells for surface CD41 and CD9 (Pharmingen, San Diego, CA) and glycophorin A (Immunotech, Brea, CA) employed fluorescein isothiocyanate-conjugated antibody per the manufacturer's recommendations. Appropriate fluorochrome-conjugated isotype matched antibody controls were used at concentrations identical to the corresponding experimental antibodies. Flow cytometric analysis was performed on either a FACScan or FACsCalibur system using Cellquest software (Becton Dickinson, San Jose, CA). Three separate cytometric analyses were performed and a representative one has been shown in Fig 1, 2 and Fig 3.

### Immunoblot Assays

Whole cell lysates were prepared by solubilizing 10^7^ cells per ml in lysis buffer (62.5 mM Tris pH 6.8, 2% SDS, 25% glycerol, 1% DTT and .01% BPB) containing 1∶100 of PMSF, NA_3_OV_4_, a protease cocktail inhibitor (#P7626, S6508, P8340, Sigma; St. Louis MO), vortexed, boiled for 10 min and stored at −80°C. Protein samples were run on 10% polyacrylamide gels and transferred to nitrocellulose membranes. Equivalent lane loading was confirmed by Ponceau S solution staining of membranes. Membranes were blocked in 5% nonfat milk/TBST for 1 hour. Except for PhosERK (Abcam, Cambridge, MA), Egr-1(C-19), Fli-1(C-19), Eklf(H-265), C-myb(C-19) PhosERK(phosphoS729) and GAPDH (FL-335) primary antibodies were purchased from Santa Cruz Biotechnology Inc. (Santa Cruz, CA). These were used at dilutions of (1∶250, 1∶200, 1∶200, 1∶300, 1∶300 and 1∶5000) respectively and blocked over night at 4°C. The secondary antibodies were HRP-conjugated goat anti-mouse or goat anti-rabbit (Bio-Rad, Hercules, CA), and were used at a 1∶5000 dilution and blocked in 5% NFDM/TBST. ECL chemiluminescent reagent (Amersham, Piscataway, NJ)was used for detection of all antibodies.

### Nucleofection and PCR

CD34+ cells 6.25×10^5^/ml in StemSpan SFEM media with 40 ng/ml SCF, 100 ng/ml TPO and 3 ng/ml IL-3 were plated and grown for 2 days. Cells were nucleofected according to the AMAXA manufacturer's protocol using 3 µg of non-sense or Si (Ambion S11102 for PKCεsi). Cells were incubated for 2 hours and then they were treated with vehicle or 25 nM IDB and were harvested 24 hr after treatment. Following harvest, cells were washed twice with PBS and an equal number of cells per tube were used to extract total RNA using TRIZOL as per the manufacturer's protocol (Invitrogen, Carlsbad, CA). The RNA was quantitated by nano drop ND-1000 (Thermo Fisher Scientific, Lafayette, CO), normalized and 50 ng was reverse-transcribed using M-MLV RT according to manufacturer's protocol (Invitrogen). PCR was performed using 1 µl of cDNA template in a 25 µl reaction volume with Qiagens HotStarTaq Master Mix Kit (Qiagen, Valencia, CA). All primers were designed and purchased from Integrated DNA Technologies (Coralville, Iowa). CD9 forward (5′-ACT GTT CTT CGG CTT CCT CTT GGT-3′) and reverse (5′-CAC TGC GCC GAT GAT GTG GAA TTT-3′) primers were amplified 30 cycles at 61°C, PKCε forward (TTC ACG GTT CTA TGC TGC CAG AGG T) and reverse (AGG GTG GTC TGA TCT TCT TCT GCT) primers were amplified at 35 cycles at 59. Housekeeper GADPH primers were utilized for equal loading and amplified at 32 with an annealing temperature of 48°C.

Quantitative real-time RT-PCR was performed using TaqMan one-step RT-PCR master mix kit (PE Applied Biosystems/Life Technologies, Grand Island, NY) with probes and primers using the SYBR green core reagent kit on an ABI 7700 sequence detection system. The expression level of human 18S RNA was determined by a predeveloped mixture of TaqMan probe and primers (PE Applied Biosystems) and used for normalization. All PCRs were performed in triplicate.

### Animal Studies

Female BALB/c mice (19–21 g) were purchased from Charles River Laboratories (Wilmington, MA) and housed in a sterile environment. All animal studies were carried out in accordance with the *Guide for the Care and Use of Laboratory Animals* (National Academy Press, Washington, DC, 1996) and the protocol was approved by the Institutional Laboratory Animal Care and Use Committee of The Ohio State University. Mice received whole body irradiation with varying doses of 6 MV X-rays using a Siemens linear accelerator (LINAC) (Siemens Medical Systems, Inc, Malvern, PA) in The Ohio State University Medical Center, Department of Radiation Oncology. Groups of up to 20 animals were irradiated simultaneously in sterile mouse cages. The cage was placed on a tissue equivalent block of Cerrobend and irradiated from the bottom up. This ensured uniform irradiation of the mice and no buildup (skin sparing) effects from the megavoltage photon beam. The radiation procedure took ∼5 to 10 min per cage.

In Study 1, mice were irradiated with a single dose of 6 MV X-rays (2Gy, 4Gy or 6Gy). For each radiation dose, there were three test groups of 16 mice each. Group 1: irradiated controls that received an intraperitoneal (i.p.) injection of sterile water with 1% DMSO; Group 2: IDB (360 µg/kg b.w.) 3 hrs prior to X-irradiation. Four animals per group were euthanized per week, for a total of four weeks. There were two baseline control groups of 4 mice each. Group 1: sterile water containing 1% DMSO only; and Group 2: IDB alone.

In Study 2, mice were irradiated with a single 6 or 8 Gy dose of X-rays. For each irradiation dose, there were three test groups of 10 mice each. Group 1: irradiated control animals that received sterile water i.p. with 1% DMSO; Group 2: IDB (720 µg/kg b.w.), administered i.p. 3 hrs prior to X-ray irradiation; and Group 3: IDB (1080 µg/kg b.w.), administered 3 hrs prior to X-irradiation. For the 6 Gy dose, mice were euthanized at 14 days post-irradiation and analyzed as described below. For the 8 Gy dose, mice were euthanized at 21 days post-irradiation.

In study 3, mice were irradiated with 6 Gy of X-rays. Group 1: baseline controls of 5 mice that received sterile water i.p. with 1% DMSO.; Group 2: irradiated animals that received sterile water with 1% DMSO 24 hrs after irradiation; Group 3: IDB (1080 µg/kg b.w.), administered i.p. 24 hrs after irradiation. Mice were euthanized at 14 days post-irradiation and analyzed as described below.

Animals were anesthetized with 2% isoflurane and bled via the retro-orbital sinus. Blood samples (100 µL) were collected from each animal and placed in 0.25 mL Minicollect® K3EDTA blood collection tubes (Greiner Bio-One, Monroe, NC). Complete blood counts (CBC) were performed on the same day. After bleeding, the animals were euthanized by exposure to 10% isoflurane, followed by cervical dislocation. The spleens and bone marrow were removed and submitted for histologic evaluation. The tissue samples were fixed in 10% buffered formalin, sections were embedded in paraffin, cut at 4 µ, stained with hematoxylin and eosin (H&E), and then examined microscopically.

### Survival study after mice received lethal doses of X-irradiation

Female BALB/c mice were irradiated with a single dose of either 8.5 or 10 Gy of 6 MV X-rays, 10 mice at the former and 20 for the latter dose. Animals received a single dose of either 360 or 1800 µg/kg b.w. of IDB 3 hrs prior to or 24 hrs after irradiation. The clinical status of the animals was monitored daily and the death dates were recorded.

### Statistical Analysis

Platelet, hemoglobin and white blood cell counts of mice between groups were compared respectively using a two-sided t-test for two-group comparisons with a Bonferroni correction if there were multiple group comparisons. To study the survival of BALB/c mice following IDB administration either before or after X-irradiation induced injury, Kaplan-Meier survival curves were plotted for each group of mice. A Log-rank test was performed to evaluate the equality of survival curves between the group receiving X-irradiation alone and the group receiving both X-irradiation and IDB treatment. The difference was considered significant if the *P*-value was <0.05. A T-test and ANOVA were performed using SPSS 19 (IBM Corp, Somers, NY), and survival data were analyzed using STATA 10.0 (Stata Corp, College Station, TX).

## Supporting Information

Figure S1 **Multiparametric immunophenotypic analysis of CD34+ cells treated with IDB.** CD34+ cells with cultured in serum free media with TPO (40 ng/ml), SCF (25 ng/ml), and 25 nM IDB for seven days. Cells were then harvested and subjected to multiparametric flow cytometric analysis. These results confirmed that the cells produced *in vitro*, following their exposure to IDB, were megakaryocytes.
(TIF)

Figure S2 **TPO/SCF supports little erythroid growth and EPO/SCF little megakaryocytic growth from CD34+ cells.** (Upper panels) CD+34 cells were cultured in serum free media with 40 ng/ml TPO and 25 ng/ml SCF for 12 days and analyzed by flow cytometry for CD41 or Glycophorin (green) versus isotype control (blue). (Lower panels) CD34+ cells were cultured in serum free media with 1 unit/ml EPO and 25 ng/ml SCF for 12 days and analyzed by flow cytometry for CD41 or Glycophorin (green) versus isotype control (blue). The results showed that cultures supported by TPO/SCF produced very few erythroid cells and those supported by EPO/SCF produced very few megakaryocytic cells.
(TIF)

Figure S3 **IDB influences cytokine specificity to promote megakaryocytic differentiation.** CD34+ progenitors were cultured for 7 days in serum free media supplemented with 25 ng/ml SCF and 1 unit/ml EPO with our without the varying concentrations of IDB. Cytospin preps were stained with either Wright's or immunocytochemical staining with anti-glycophin A (GPA) or anti-CD41, as indicated. The addition of IDB to erythroid cultures lead to a dose dependent decrease in GPA-positive erythroid cells and an increase in CD41-positive megakaryocytic cells.
(TIF)
