# Supplementary figures and images for: Unique In Vitro and In Vivo Thrombopoietic Activities of Ingenol 3,20 Dibenzoate, A Ca++-Independent Protein Kinase C Isoform Agonist
Source: PLoS One. 2012 Dec 21;7(12):e51059. doi: 10.1371/journal.pone.0051059 (PMC3528756; doi:10.1371/journal.pone.0051059)

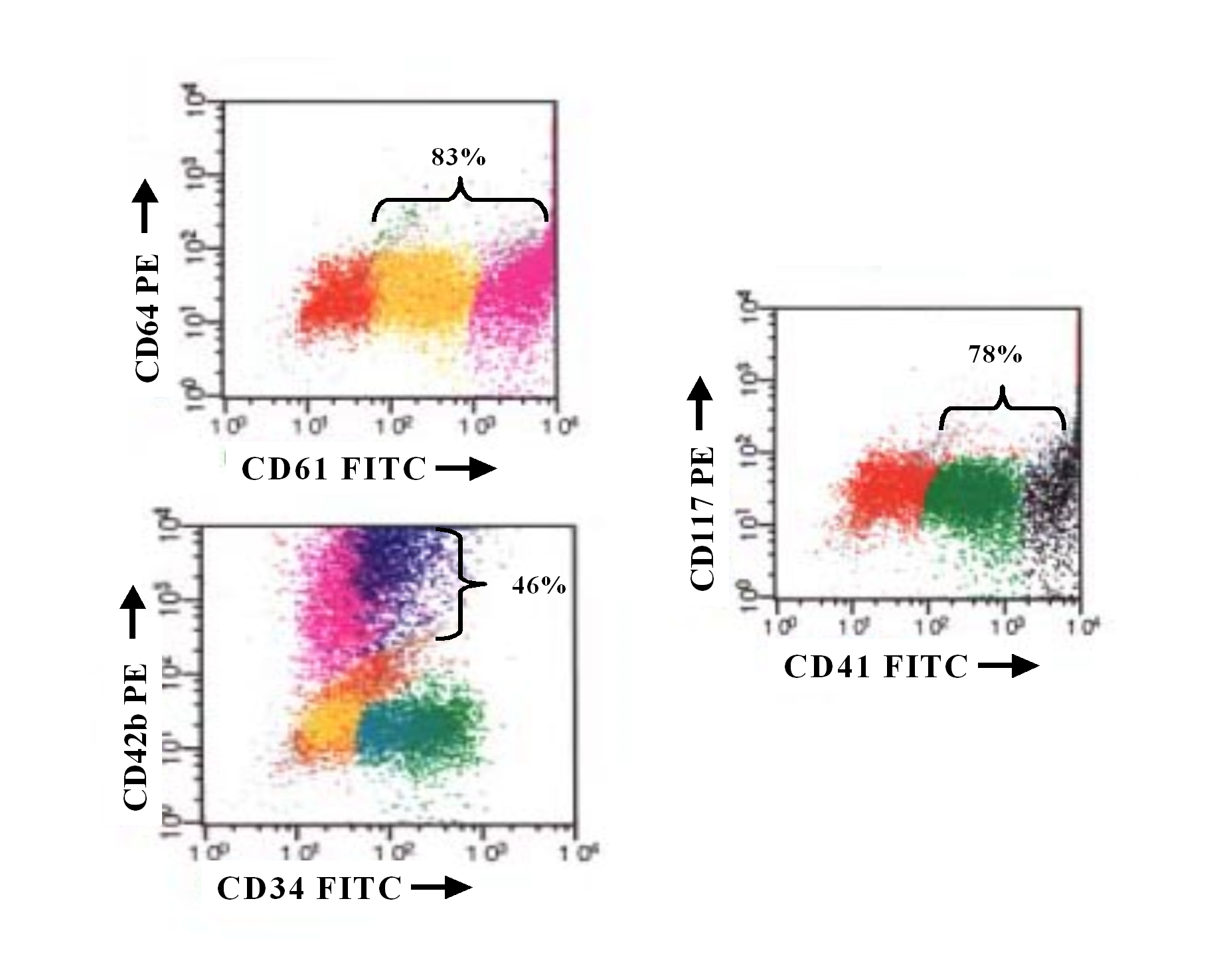

Supplement: Figure S1 — Multiparametric immunophenotypic analysis of CD34+ cells treated with IDB. CD34+ cells with cultured in serum free media with TPO (40 ng/ml), SCF (25 ng/ml), and 25 nM IDB for seven days. Cells were then harvested and subjected to multiparametric flow cytometric analysis. These results confirmed that the cells produced in vitro, following their exposure to IDB, were megakaryocytes. (TIF) [file pone.0051059.s001.tif]

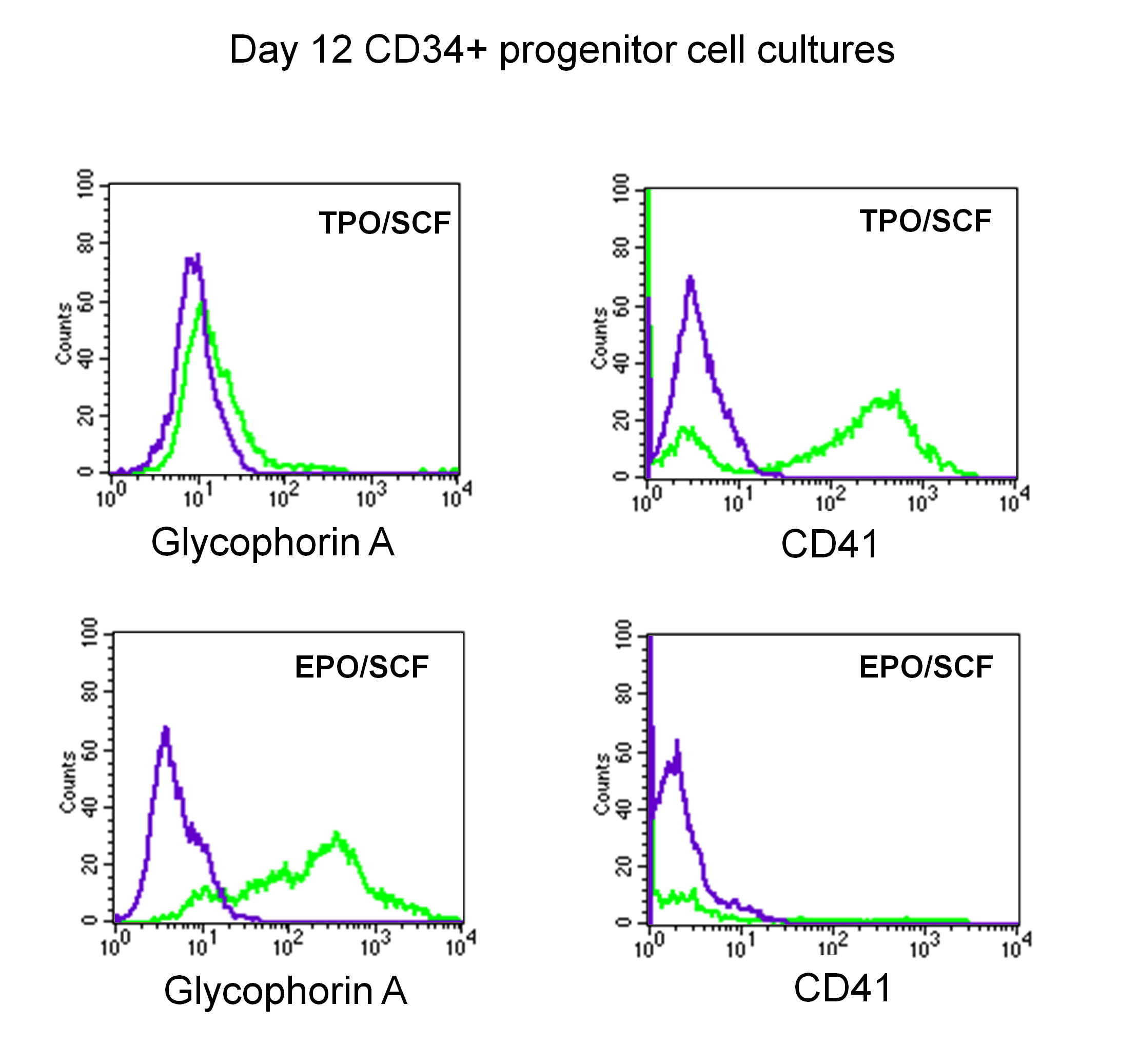

Supplement: Figure S2 — TPO/SCF supports little erythroid growth and EPO/SCF little megakaryocytic growth from CD34+ cells. (Upper panels) CD+34 cells were cultured in serum free media with 40 ng/ml TPO and 25 ng/ml SCF for 12 days and analyzed by flow cytometry for CD41 or Glycophorin (green) versus isotype control (blue). (Lower panels) CD34+ cells were cultured in serum free media with 1 unit/ml EPO and 25 ng/ml SCF for 12 days and analyzed by flow cytometry for CD41 or Glycophorin (green) versus isotype control (blue). The results showed that cultures supported by TPO/SCF produced very few erythroid cells and those supported by EPO/SCF produced very few megakaryocytic cells. (TIF) [file pone.0051059.s002.tif]

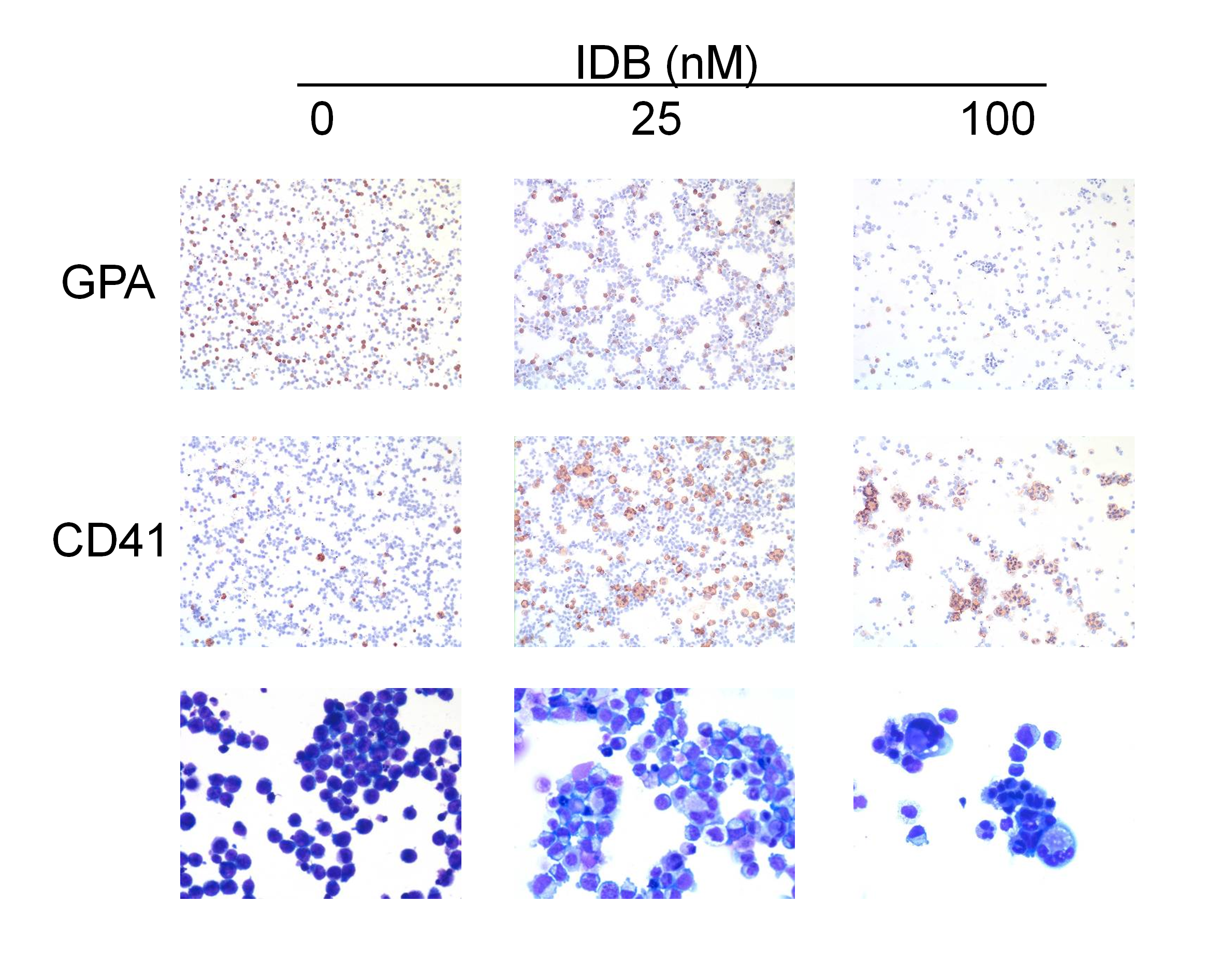

Supplement: Figure S3 — IDB influences cytokine specificity to promote megakaryocytic differentiation. CD34+ progenitors were cultured for 7 days in serum free media supplemented with 25 ng/ml SCF and 1 unit/ml EPO with our without the varying concentrations of IDB. Cytospin preps were stained with either Wright's or immunocytochemical staining with anti-glycophin A (GPA) or anti-CD41, as indicated. The addition of IDB to erythroid cultures lead to a dose dependent decrease in GPA-positive erythroid cells and an increase in CD41-positive megakaryocytic cells. (TIF) [file pone.0051059.s003.tif]
